# CHD4 acts as a prognostic factor and drives radioresistance in HPV negative HNSCC

**DOI:** 10.1038/s41598-024-58958-z

**Published:** 2024-04-09

**Authors:** Fabian Geyer, Maximilian Geyer, Ute Reuning, Sarah Klapproth, Klaus-Dietrich Wolff, Markus Nieberler

**Affiliations:** 1 Department of Oral and Maxillofacial Surgery, Klinikum Rechts der Isar der Technischen Universität München, 81675 Munich, Germany; 2 Clinical Research Unit, Department of Obstetrics and Gynecology, Technical University of Munich, 81675 Munich, Germany; 3 Institute of Experimental Hematology, School of Medicine, Technische Universität München, 81675 Munich, Germany

**Keywords:** Head and neck cancer, Oral cancer

## Abstract

Despite great efforts in improving existing therapies, the outcome of patients with advanced radioresistant HPV-negative head and neck squamous cell carcinoma (HNSCC) remains poor. The chromatin remodeler Chromodomain helicase DNA binding protein 4 (CHD4) is involved in different DNA-repair mechanisms, but the role and potential in HNSCC has not been explored yet. In the present study, we evaluated the prognostic significance of CHD4 expression using in silico analysis of the pan-cancer dataset. Furthermore, we established a monoclonal HNSCC CHD4 knockdown cell clone utilizing the CRISPR/Cas9 system. Effects of lower CHD4 expression on radiosensitivity after increasing doses of ionizing radiation were characterized using clonogenic assays and cell numbers. The in silico analysis revealed that high CHD4 expression is associated with significant poorer overall survival of HPV-negative HNSCC patients. Additionally, the knockdown of CHD4 significantly increased the radiosensitivity of HNSCC cells. Therefore, CHD4 might be involved in promoting radioresistance in hard-to-treat HPV-negative HNSCC entities. We conclude that CHD4 could serve as a prognostic factor in HPV-negative HNSCC tumors and is a potential target protein overcoming radioresistance in HNSCC. Our results and the newly established cell clone laid the foundation to further characterize the underlying mechanisms and ultimately use CHD4 in HNSCC therapies.

## Introduction

Head and neck squamous cell carcinoma (HNSCC), including oral, pharyngeal, and laryngeal malignancies, are the sixth most common cancer type worldwide^1^. Oral squamous cell carcinoma (OSCC) is the most frequent single entity of oral cancer, representing the largest group of head and neck cancers^2^. Presently, the treatment options for HNSCC predominantly include surgical resection, chemotherapy, and radiotherapy, either in combination or as a single therapy^3^. Different carcinogenic factors are known to date in HNSCC patients, particularly alcohol and tobacco. In recent years, infection with high risk human papilloma viruses (HPV) is gaining importance as the incidence of HPV-positive HNSCC has risen substantially^4,5^. However, patients afflicted with HPV-positive tumors have better overall survival and show remarkably higher radiosensitivity compared to HPV-negative tumors^6,7^. Despite great efforts in improving existing therapies, the 5-year overall survival rate of advanced HNSCC remains low^8,9^. Therefore, the identification of new target proteins plays a huge role in cancer research to enhance the effectiveness of commonly known treatment options.

One of the lethal DNA damages taken advantage of in cancer therapy are DNA double-strand breaks (DSB) induced by ionizing radiation (IR)^10^. Genomic stability and cellular viability are dependent on functioning mechanisms to repair DSBs. Chromatin remodeling is an essential factor for initiating, propagating, and terminating effective DNA repair^11^.

Chromodomain helicase DNA binding protein 4 (CHD4, also known as Mi-2β), a highly conserved and adenosine triphosphate (ATP) dependent chromatin remodeling factor, is a core subunit of the nucleosome remodeling and deacetylase (NuRD) complex^12,13^. CHD4 is linked to multiple important functions in cancer cells, such as cell cycle regulation, cell differentiation, and DNA repair^14–19^. Through controlling homologous recombination repair to maintain genome stability and initiating the epigenetic suppression of different tumor suppressor genes, CHD4 is implied to have oncogenic functions^20^. High expression levels of CHD4 are associated with poor prognosis in various cancer types^20–24^ and was shown to correlate with radioresistance in patients afflicted with colorectal cancer^20^. In response to oxidative stress or ionizing radiation, CHD4 is important in maintaining genome integrity by regulating signaling pathways and DNA damage repair^19,25,26^. CHD4 is rapidly recruited to sites of DSBs and DNA damage through different mechanisms. In association with Poly(ADP-ribose)-Polymerase 1 (PARP1), CHD4 is relocated to DNA damage sites along with the NuRD complex creating a repressive chromatin structure to prevent the transcription of damaged genes^20,27^. RING finger ubiquitin ligase 8 (RNF8) also recruits CHD4 to damaged DNA structures independently from other NuRD subunits. This supports the assembly of additional DNA repair factors, such as BRCA1 and RNF168^19^. In cases of DNA damage, ATM and ATR, acting as DNA damage response (DDR) kinases, phosphorylate CHD4, indicating its involvement in different mechanisms of cell survival and DNA repair^22,28,29^. Lastly, Qi et al. demonstrated the collaboration between acetyltransferase p300 and CHD4 in facilitating DSB repair. Both may function cooperatively at DSB sites. Their transient knockdown impaired homologous recombination (HR) repair and suppressed the recruitment of replication protein A (RPA), a key protein for HR. Ablation of these proteins sensitized cells to laser and the anti-cancer drug etoposide and decreased DSB repair^30^. Similar effects have also been reported in different cancer types^22,31^. However, the understanding of the roles of CHD4 in HNSCC cells remains elusive.

In this study, we aimed to evaluate the prognostic relevance of CHD4 in HNSCC patients and establish stable CHD4-knockdown HNSCC cells using the CRISPR/Cas9 gene editing system, to determine, if CHD4 could be identified as a possible target protein driving radioresistance, proliferation and colony formation ability in irradiated HNSCC cells.

## Results

### High CHD4 gene expression is associated with worse overall survival in HPV-positive HNSCC

**Figure 1:**
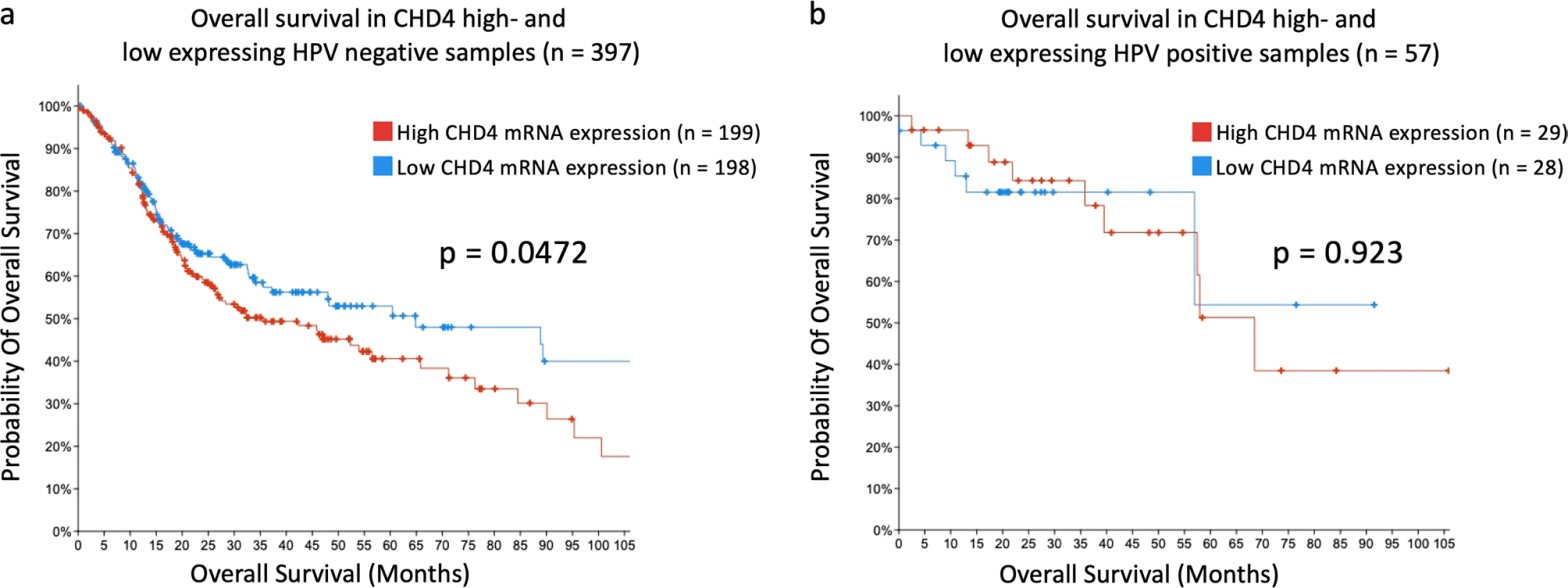
Impact of CHD4 gene expression on overall survival of HPV negative and positive patients. (**a**, **b**) The association of high and low CHD4 expression levels on overall survival of HPV-negative (n = 397) and HPV-positive (n = 57) HNSCC patients, generated from the cBioPortal HNSCC TCGA PanCancer Atlas dataset. Groups were divided based on the median and presented using Kaplan–Meier method (log-rank test, *p* = 0.0472 and *p* = 0.923).

In order to investigate the role of CHD4 in patients with HNSCC, we systematically analyzed tumor tissue of HNSCC patients included in the Cancer Genome Atlas (TCGA) HNSCC cohort concerning CHD4 gene expression levels and HPV status. As the HPV infection status has great influence on radiosensitivity, pathogenesis and gene expression with a lower overall mutation rate in HPV-positive HNSCC, we separated the tumor samples into two groups^1,7^. The CHD4 mRNA expression in HPV-negative and HPV-positive HNSCC samples were defined high and low based on the median z-score. In the HPV-negative cohort, patients with low CHD4 expression were defined with a z-score of ≤ 0.21 and with high CHD4 levels with a z-score of > 0.21. The Kaplan–Meier analysis in the HPV-negative HNSCC cohort revealed a significant (*p* = 0.0472) impact of high CHD4 expression on the overall survival probability of patients. The median overall survival (OAS) of this group was 35.47 months, while low CHD4-expressing samples showed a median OAS of 64.83 months (Fig. 1a). To explore, if a correlation exists between CHD4 expression and the HPV status of HNSCC patients, we analyzed the HPV-positive HNSCC cohort for OAS. Again, groups were selected based on the median CHD4 expression and the outcome of patients was analyzed using the Kaplan–Meier method. Patients with high CHD4 expression, defined as z-score > − 0.03, showed a median OAS of 69.64 months. Samples with low CHD4 expression, defined as a z-score ≤ − 0.03, showed a median OAS of 68.48 months (Fig. 1b). Therefore, CHD4 expression levels had no significant (*p* = 0.923) impact on the prognosis of patients in the HPV-positive HNSCC cohort. Taken together, these data suggest CHD4 as a potential prognostic factor for HPV-negative HNSCC patients.

### Genomic knockdown of CHD4 in HN cells

**Figure 2:**
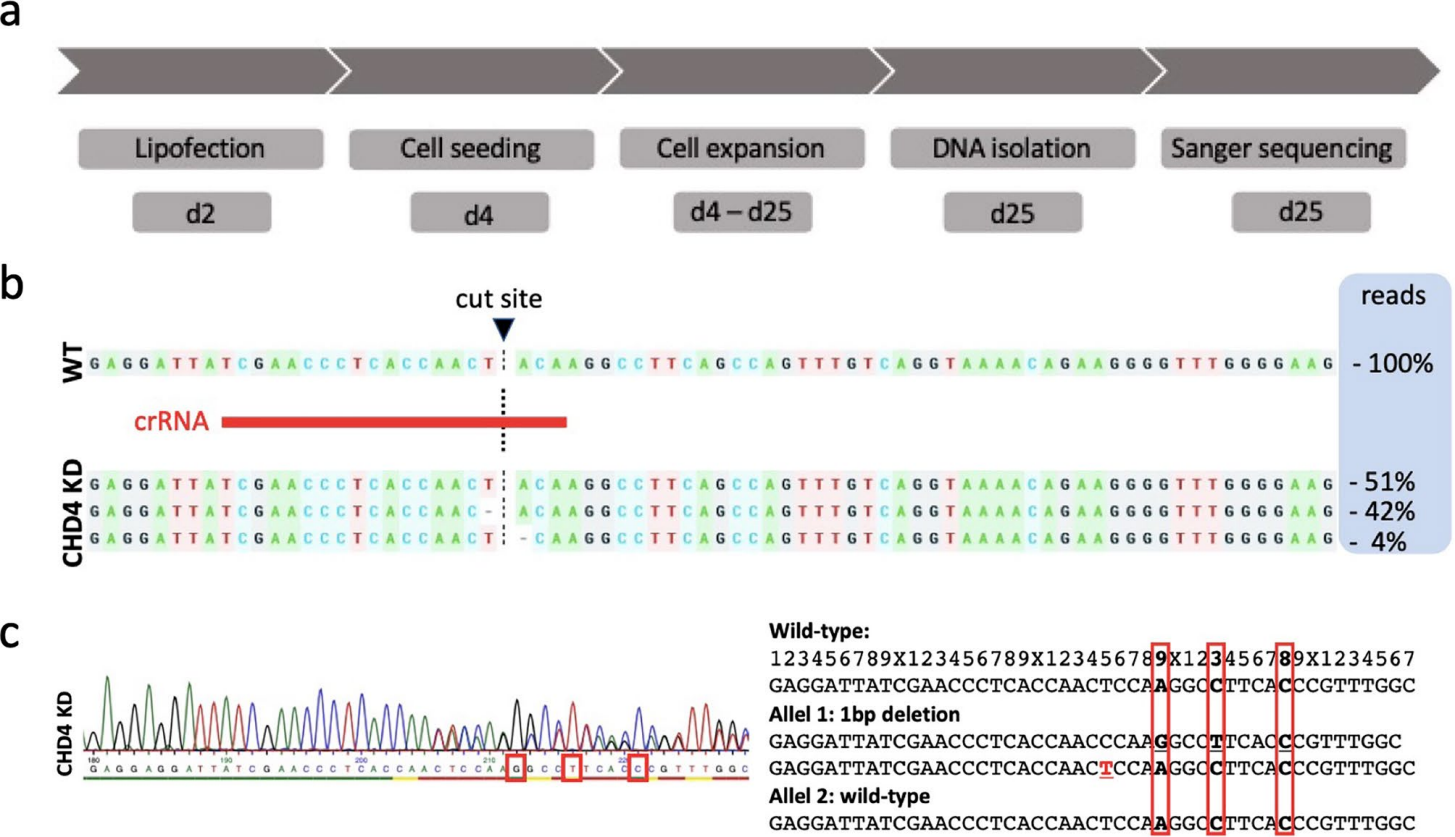
CRISPR/Cas9-mediated CHD4 knockdown in HN cells. (**a**) Complete workflow for the generation of HN CHD4 KD cells. (**b**) Sequencing traces of the target site after analysis of sequencing data with Synthego ICE online tool in the CHD4 locus of HN cells. Cas9 cut site is marked with the dotted line, crRNA binding site is shown in red. The charts represent percentages of reads of individual INDELs within the WT compared with CHD4 KD clones. The sequence reads were aligned to the WT reference allele and percentages of reads are shown for HN WT and HN CHD4 KD clones. CHD4 KD clones show INDELs around the cut site. (**c**) Chromatogram after Sanger sequencing and manual alignment with HN WT reference allele.

For generating monoclonal HN CHD4 knockdown (KD) cells and further analyze the role of CHD4 in vitro, we optimized and adapted the CRISPR/Cas9 workflow to the here used HN cell line (Fig. 2a). Before conducting CRISPR/Cas9 editing, high CHD4 expression of the used HN cells was confirmed in comparison to the HNSCC cell line BHY (*ACC 404)* (Figure S2). Initially, three different guide sequences were tested and the best performing CRISPR RNA (crRNA) was chosen. Editing efficiency was highly dependent on the transfected amount of RNP complexes and cell confluency, resulting in up to 73% (mean 67.33%) insertion and deletion mutations (INDEL), as we reported previously^32^. Given the high Indel%, a single crRNA approach with limiting dilution was possible. First DNA isolation of the individual clones was achievable after approximately 3 weeks to proceed with PCR and clone screening by Sanger sequencing. At first, the initial chromatograms of clones with overlapping reads downstream the crRNA binding site were considered putative KO or KD clones and were then further analyzed with the Synthego ICE or TIDE online tool. The resulting sequencing traces of the selected HN CHD4 KD clone after ICE analysis are shown in Fig. 2b. The CRISPR-edited clones were aligned to a confirmed HN wild type (WT) sequence to determine CRISPR-induced INDELs. Aligned to the reference WT allele, INDEL quantification represents percentages of total reads of individual INDELs within HN CHD4 KD. Around the cut site, edited alleles in the CHD4 KD sequence are visible, compared to no INDELs in the WT sequence. Allele 1 shows an unedited WT sequence representing 52% of total reads, whereas allele 2 presents a one base pair (bp) deletion upstream the cutting site and a one bp deletion downstream the cut site, representing 42% and 4% of total reads respectively (Fig. 2b). These data indicate a CHD4 KD clone with one WT allele and a knocked-out second allele with a frameshift mutation. For further investigation, the sequences around the suspected cut site were aligned manually (Fig. 2c). It was tested, which assumed insertions or deletions would result in identical base calls at determined positions (red boxes Fig. 2c) compared to the WT sequence. Again, it revealed one edited allele resulting in a frameshift mutation, and one WT allele.

**Figure 3:**
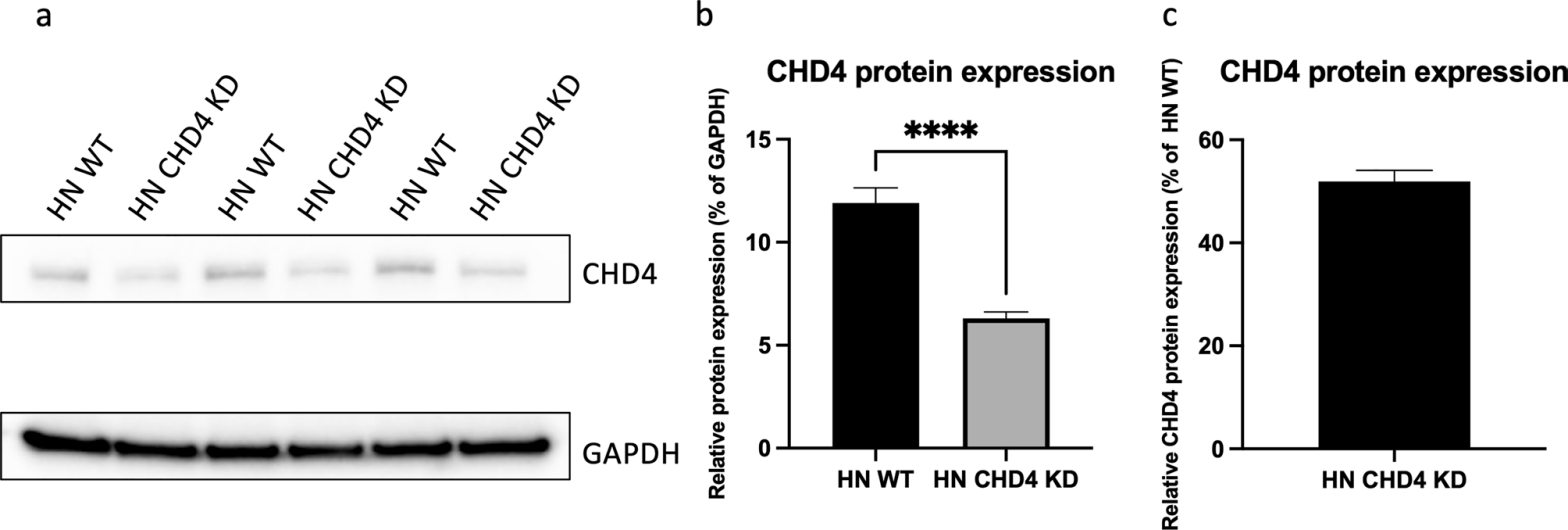
CHD4 protein expression in HN CHD4 KD cells. (**a**) Protein expression of CHD4 in HN WT and HN CHD4 KD cells as determined by Western blot analysis. GAPDH was used as control for equal loading and blotting efficiency. Protein samples of three different experiments were loaded onto one gel and blotted. The figure displays a cropped blot, the original blot is presented in Supplementary Figure S1. (**b**) Densitometric analysis of the relative CHD4 expression in % of GAPDH of HN WT cells in comparison to HN CHD4 KD cells (unpaired *t*-test, ****p < 0.0001). (**c**) CHD4 expression in % of HN CHD4 KD cells directly compared to HN WT cells. Bars represent mean ± SEM. N = 4.

After genomic analysis, Western blot analysis was performed to verify successful gene editing on the protein level and to investigate the impact of the genomic mutations on the CHD4 protein expression in the generated HN CHD4 KD cells (Fig. 3a). Densitometric analysis was performed and relative CHD4 expression levels were normalized to Glyceraldehyde-3-phosphate dehydrogenase (GAPDH) expression levels. As expected, there is a significant (*p* < 0.0001) drop in CHD4 protein concentration in the HN CHD4 KD cells compared to the unedited HN WT control cells (Fig. 3b). Directly compared to HN WT, the edited cells show 51.91% (± 1.08%) of assembled CHD4 protein, supporting our sequencing results (Fig. 3c). Maintenance of knockdown efficiency in HN CHD4 KD cells was confirmed with continued cell passage (Figure S3).

In summary, we demonstrated a successful approach to CRISPR editing of the human CHD4 locus and validated the generation of stable human HN CHD4-KD cells.

### Characterization of HN CHD4 subclones

After cell expansion to sufficient numbers, the *Polymerase chain reaction (PCR)* products of 30 individual single cell clones were analyzed by Sanger sequencing. Both alleles were checked for putative target mutations in the CHD4 sequence downstream the Cas9 cut site, especially frameshift mutations. We split the occurred mutations in different groups, as presented in Table 1. Frameshift mutations were defined as insertions or deletions in which the total number of nucleotides is not divisible by 3, non-frameshift mutations accordingly. Only 4 out of 30 cell clones showed the WT sequence of CHD4 with no mutations at all, underlining the adequate DNA cleavage of our CRISPR/Cas9 set-up (Table 1). The largest group represents cells with one unedited WT allele and one allele with frameshift mutation. Across all edited alleles of the 30 investigated cell clones, no large insertions or deletions > 20 bp could be found. Interestingly, despite the high editing efficiency of our chosen guide sequence, no complete CHD4 knockout with according mutations in both alleles was observed. Therefore, CHD4 might be a crucial component for HN cell viability and expansion, especially in colonies arising from single cells.

**Table 1:** Characterization of HN CHD4 subclones.

Allele 1/ allele 2	WT/WT	WT/ frameshift	WT/Non-frameshift	Non-frameshift/ frameshift	Non-frameshift/non-frameshift
Clone number	CHD4 #8 CHD4 #2.2 CHD4 #2.12 CHD4 #2.13	CHD4 #1 CHD4 #2 CHD4 #3 CHD4 #5 CHD4 #6 CHD4 #7 CHD4 #12 CHD4 #14 CHD4 #18 CHD4 #2.17 CHD4 #2.21	CHD4 #9 CHD4 #10 CHD4# 2.4 CHD4 #2.6 CHD4 #2.11	CHD4 #2 CHD4 #13 CHD4 #15 CHD4 #2.5 CHD4 #2.14 CHD4 #2.20 CHD4 #2.22	CHD4 #4 CHD4 #11 CHD4 #2.1
Total	4/30	11/30	5/30	7/30	3/30

All HN CHD4 subclones were analyzed using Sanger sequencing. The sequences were then further examined with TIDE and Synthego ICE for putative mutations. We divided the HN CHD4 subclones into different groups based on the analysis: Frameshift mutations defined as INDELs not divisible by three, non-frameshift mutations as INDELs divisible by three. Both alleles of each clone were analyzed. N = 30.

### Effects of irradiation on cell viability

**Figure 4:**
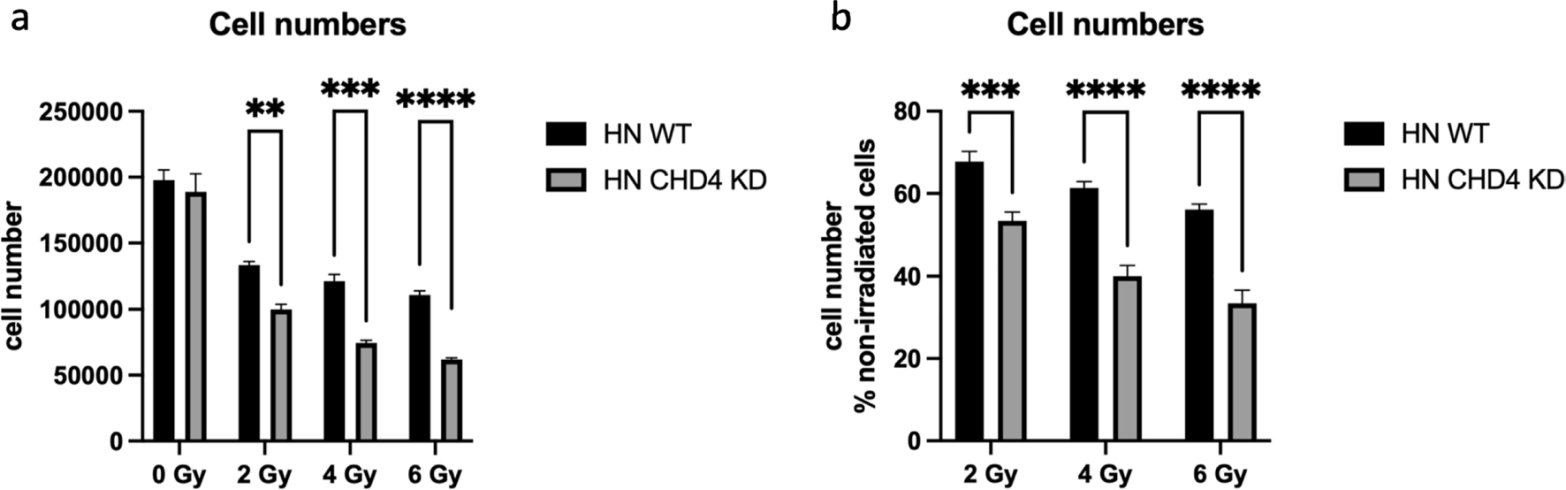
Influence of irradiation on numbers of viable cells. (**a**) The absolute cell numbers of non-irradiated HN WT and HN CHD4 KD and respective cell lines irradiated with 2, 4, and 6 Gy, were compared 48h after irradiation (unpaired *t*-test, **p = 0.0041, ***p = 0.0001, ****p < 0.0001). (**b**) Radiation effects on absolute cell numbers of each cell line were analyzed 48 h after radiation treatment and are depicted as percentage to the respective untreated control cells (100%) (unpaired *t*-test, ***p = 0.0009, ****p < 0.0001). Bars represent mean ± SEM. N = 5.

48 h after irradiation, adherent HN cells were detached and viable cells counted upon trypan blue exclusion in order to analyze the influence of radiation treatment on proliferation of HN WT cells compared to CHD4 KD cells. No significant differences in absolute cell numbers were observed in the untreated control cells, indicating a similar basic proliferation rate in both cells (Fig. 4a). Cell irradiation led to a dose-dependent decrease in cell numbers of both cell variants. As shown in Fig. 4a, HN WT cells were more radioresistant across all applied radiation doses compared to the HN CHD4 KD cells, as indicated by a significantly higher absolute cell count. Figure 4b shows the absolute cell numbers of non-irradiated cells set to 100% and the radiation effects calculated respectively. A significant difference between both cell types was also detected at doses of 2, 4, and 6 Gy, respectively, when compared to untreated cells (Fig. 4b). Hence, while different CHD4 expression levels had limited effects on cell proliferation in untreated HN cells, lower CHD4 levels correlated with enhanced radiosensitivity.

### Effects of irradiation on colony formation

**Figure 5:**
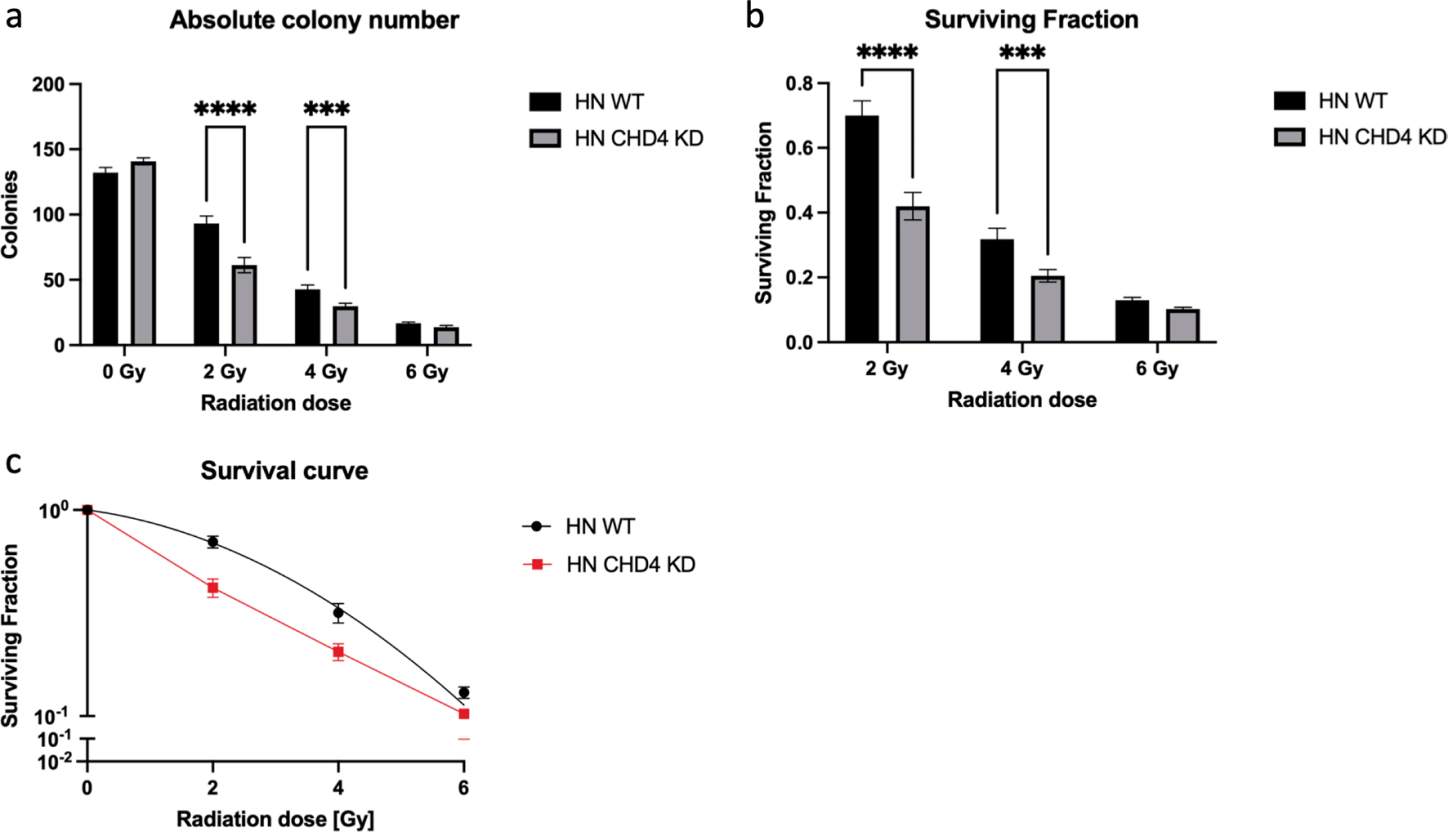
Influence of irradiation on colony forming ability of HN cells. (**a**) The absolute number of colonies formed by untreated HN WT and HN CHD4 KD cells and respective cells irradiated with 2, 4 and 6 Gy, respectively, were counted and compared. Colonies were defined as cell clusters containing ≥ 50 cells (unpaired *t*-test, ****p < 0.0001, ***p = 0.0002). (**b**) Calculated surviving fraction of colonies after radiation treatment. HN WT compared to HN CHD4 KD colonies (unpaired *t*-test, ****p < 0.0001, ***p = 0.0002). (**c**) Radiation survival curves of HN WT (black) and HN CHD4 KD (red) cells. Survival data were fitted to linear-quadratic model. Bars represent mean ± SEM. Experiments were conducted in duplicates. N = 5.

In order to evaluate HN cell survival based on the ability of single cells to grow into colonies and also to further characterize the influence of CHD4 expression on radioresistance, colony formation assays were performed. The clonogenic ability decreased in all cells following radiation treatment with increasing doses as expected, when considering absolute colony numbers, as well as the calculated surviving fractions. However, no significant difference was observed in the untreated control cells (Fig. 5a, b). HN CHD4 KD cells showed higher radiosensitivity compared with HN WT cells. In particular at doses of 2 or 4 Gy, a significant difference was detectable. When further approaching the lethal radiation dose of HN cells, a lower effect on clonogenic ability at 6 Gy could be seen (Fig. 5a, b). Lastly, a dose-dependent survival curve of both cell variants is presented (Fig. 5c). The survival curve was fitted to the linear-quadratic model of Y = exp( − 1*(A*X + B*X^2^)), where Y is the fraction of cells surviving radiation treatment, X the dose of radiation and A and B the linear and quadratic components of cell killing. In sum, the data derived from colony formation assays again suggests the positive impact of low CHD4 expression levels on increased radiosensitivity of HN cells, while CHD4 had no influence on colony forming ability in untreated cells.

## Discussion

Radiotherapy is a commonly known treatment regimen for HNSCC patients besides surgical resection and chemotherapy^3^. However, as higher radiation doses generate more DNA damage, treatment efficacy is often accompanied with serious short- and long-term side effects on the surrounding healthy tissue and therefore has its limitations in escalating dose^33,34^. Tumor cells frequently develop different strategies to evade targeted therapies, leading e.g. to radioresistance and, consequently, tumor progression with poor patient outcome^35^. Identifying possible target proteins that drive resistance to radiation treatment is thought to clinically benefit cancer patients, especially in the vulnerable field of the head and neck. In this study, we aimed at evaluating the prognostic relevance of CHD4 in HNSCC, establishing stable CHD4 KD HNSCC cells utilizing the CRISPR/Cas9 gene editing system and further evaluate the influence of different CHD4 expression levels on proliferation and colony forming ability of tumor cells exposed to increasing doses of IR.

In silico data analysis revealed the negative impact of high CHD4 gene expression on OAS of HPV-negative HNSCC patients. Additionally, we showed the successful CRISPR/Cas9-mediated gene editing of the human CHD4 locus in HPV negative HN cells and CHD4-KD led to increased radiosensitivity.

HNSCC, based on etiology, can be divided into two main subtypes: HPV-related (HPV-positive) and HPV-unrelated (HPV-negative)^36^. While HPV-positive HNSCC usually tends to arise in younger patients and is more sensitive to treatment with better survival rates, in contrast, treatment of HPV-negative HNSCC is more challenging. At 3 years, overall survival rates are estimated at 82% in locally advanced HPV-positive HNSCC compared to 57% in locally advanced HPV-negative HNSCC^37,38^. Additional preclinical and clinical data recommend a different therapeutic strategy of the two subtypes and consequently different HNSCC clinical trials are based on HPV infection status recently^39^. As HPV alters gene expression and has a great influence on radiosensitivity, especially in hard-to-treat HPV-negative HNSCC, more effective and toxicity-acceptable treatment regimen are urgently needed^1,7^.

Li et al. recently linked high CHD4 expression in OSCC to prognosis with reduced OAS in OSCC patients. However, as mentioned above, HPV-positive and -negative HNSCC tumor entities should be evaluated separately. As our analysis shows, high CHD4 gene expression is of prognostic significance in HPV-negative HNSCC patients, but not in HPV-positive tumors. Therefore, we here propose CHD4 as a novel prognostic factor in HPV-negative HNSCC patients. Besides HNSCC, previous studies suggested CHD4 as a prognostic marker in papillary thyroid cancer and low CHD4 expression had a beneficial effect on OAS and lower disease recurrence rates in patients afflicted with colorectal cancer^24,40,41^.

To evaluate the impact on radiosensitivity, we established a stable CHD4 KD clone. We showed the successful CRISPR/Cas9-mediated gene editing of the human CHD4 locus in the HNSCC cell line HN. The CHD4 KD resulted in significantly reduced CHD4 protein expression. While gene editing using CRISPR/Cas9 remains hugely challenging in human primary cells due to their short replicative time span, the CRISPR system is successfully utilized in a wide range of immortalized cells, especially cancer cell lines^42,43^. However, despite high editing efficiencies and excessive screening of single cell clones, no complete CHD4 KO-clone was achieved.

Regarding the proliferation of untreated cells, we did not observe significant differences between the HN WT and CHD4 KD cells. However, complete KO of CHD4 seemed to prevent HN cell expansion. CHD4 is known to contribute to the control of cell cycle progression, especially needed in the stage of single cell expansion. Together with NuRD, CHD4 controls p53 deacetylation, hence being an important regulator of the G_1_/S transition^44,45^. The activity of p53 is restricted by CHD4/NuRD-mediated deacetylation, enabling the progression of cells through the G_1_/S cell cycle boundary. In cases of CHD4 deficiency, p53 becomes hyperacetylated and hyperactive, which leads to upregulated p21 expression and ultimately G_1_/S cell cycle arrest^44^.

Additionally, it has been reported, that CHD4 and NuRD are required for cell growth in different cancer cells^20–22,31,46^. However, the literature presents ambiguous data. As Heshmati et al. showed, CHD4 KD prevented the cell growth of HL-50 (promyelocytic leukemia), K526 (chronic myelogenous leukemia), and NB-4 (acute promyelocytic leukemia) cells to a similar degree. Loss of function of CHD4 caused cell cycle arrest in the G_0_ phase. In contrast, CHD4 was not required for cell growth of normal hematopoietic cells^17^. Therefore, CHD4 might be involved in more complicated mechanisms regulating cell growth in a cell type specific manner.

When we compared the cell numbers of HN WT and HN CHD4 KD cells 48 h after irradiation with the untreated cells, HN WT showed significantly higher numbers of vital cells at all applied radiation doses, indicating that higher CHD4 expression endowed the cells with less radiosensitivity. Similar effects were observed when evaluating the colony forming ability of the two cell types. While there was no significant difference in the untreated group, CHD4 KD cells displayed significantly fewer colonies after radiation treatment, especially at irradiation doses of 2 and 4 Gy, respectively. Only at high doses of 6 Gy, no significant effect could be seen. It seems that the irradiation with 6 Gy is approaching the lethal dose for the used HN cells and the effects of different CHD4 expression is disappearing when looking at the colony forming ability. Radiation induced cell damage is a complex mechanism, especially leading to necrosis at high doses^47,48^. Different mechanisms may cooperate and therefore the actual significance of altered CHD4 expression may be lowered at higher doses. Taken together, our data suggests CHD4 as a promising and prominent druggable protein that drives radioresistance in HNSCC, particularly at common dose fractions of 2 and 4 Gy.

DNA damage-induced cell death due to IR is the basis of successful cancer radiotherapy and the negative effect of irradiation was shown in different studies for HNSCC^49–51^. However, patient outcome in HPV negative HNSCC remains poor^1,7^. In recent years, CHD4 was found to be involved in DNA damage repair through different mechanisms. Larsen et al. reported the reduced ability of cancer cells to form colonies in CHD4 KD human U2OS osteosarcoma and 293T embryonic kidney cells following ionizing radiation. Also, p21 accumulation with enhanced cell cycle delay as well as impaired assembly of DNA repair factor BRCA1 together with disruption of RNF8- and RNF168-mediated histone ubiquitylation pathways were observed. While CHD4 KD led to moderate changes in cell cycle progression in unstressed cells, the siRNA induced CHD4 KD combined with IR had a more pronounced effect. Cells showed weaker proliferation together with S-phase delay and accumulation in G_2_, a fraction ultimately arrested in G_2_^19^. Upon binding to poly(ADP-ribosyl)ated proteins (PARP), CHD4 has been shown to be recruited to PARP-dependent sites of DNA damage^27,44^. The positive impact of PARP inhibitors on radiosensitivity and impaired DSB repair has been shown in HNSCC cells in 2D and 3D models^52,53^. Additionally, PARP inhibitors and CHD4 depletion may have a synergistic effect. As Pan et al. showed, CHD4 depletion in MCF10A cells reduced HR repair, sensitized cells to DSB inducing agents, and enhanced the effect of PARP inhibitors^54^. Ultimately, Oyama et al. introduced the first-in-class SMARCA5/CHD4 inhibitor ED2-AD101 in ovarian cancer cells. A novel possible treatment approach was shown, as the inhibitor sensitized the cells to DNA damage inducing platinum agents^55^.

Our findings highlight the therapeutic relevance of CHD4, particularly in radioresistant HPV-negative HNSCC. Targeting CHD4 may enhance the efficacy of low-dose radiotherapy, thereby reducing side effects or potentially overcoming radioresistance. Our newly established stable HN CHD4 KD cells will help to further characterize the role of CHD4 in HNSCC and investigate the underlying mechanisms. This research aims to develop novel therapeutic agents and introduce CHD4 inhibitors in radioresistant HNSCC.

## Methods

### TCGA data analysis

The Cancer Genome Atlas data were accessed via cBioPortal (https://www.cbioportal.org). For our purpose, we used the Head and Neck Squamous Cell Carcinoma TCGA PanCancer Atlas dataset, which includes 523 tumor samples, to investigate the influence of different CHD4 mRNA gene expression levels on the overall survival probability of HPV-positive and HPV-negative HNSCC patients. Only patients with primary HNSCC tumors, available HPV status, and T-stage were included, reducing the total sample number to 454. The cohort was split into a HPV-negative (n = 397) and a HPV-positive (n = 57) group. Next, each group was queried regarding mRNA expression of CHD4. The mRNA expression levels in the samples were normalized relative to the mRNA contents in diploid tissue (RNA seq V2 RSEM). Lastly, the HPV positive and HPV negative cohorts were divided into two groups based on the median CHD4 expression.

### Cell line and cell culture

HN cells (cat.-nr. ACC 417, DSMZ, Braunschweig, Germany), established from a lymph node metastasis of a 60-year-old man with a moderately differentiated and HPV negative squamous cell carcinoma of the soft palate, were cultured in Dulbeccos Modified Eagle Medium (DMEM) (Sigma-Aldrich, St. Louis, Missouri, USA) supplemented with 10% (v/v) fetal bovine serum (FBS) (Gibco, Carlsbad, California, USA). All cells were maintained at 37 °C in a humified incubator with 5% (v/v) CO_2._ They were tested negative for mycoplasma contamination at periodic intervals and the growth medium was changed regularly.

### CRISPR/Cas9 gene editing and single-cell cloning

In a previous publication by us, we described the established workflow of CRISPR/Cas9-mediated knockdown of CHD4, utilizing lipofection of ribonucleoprotein complexes (RNP)^32^. Suitable custom guide sequences, targeting the human CHD4 locus on chromosome 12, were designed using the online tool CHOPCHOP (https://chopchop.cbu.uib.no)^56^ and ordered as crRNA from IDT™ (IDT, Coralville, Iowa, USA). The guide sequence used in this study was CHD4#2 (TCGAACCCTCACCAACTACA), genomic position Chr. 12 6601667. Additionally, a flanking primer-pair (fwd: GTTTCCTAGACACCTTACTGCCC; rev: CTGATGCCCCAGAACTGCCTTTG) was designed and synthesized by Eurofins Genomics, Ebersberg, Germany. RNPs were assembled together with TrueCut™ Cas9 Protein v2 (cat.-nr. A36498, Invitrogen, Darmstadt, Germany). The cells were transfected with the formed RNPs by Lipofectamine™ CRISPRMAX™ Cas9 Transfection reagent (cat-.nr. CMAX00008, Invitrogen, Darmstadt, Germany) for 48 h. After transfection, single cell cloning was performed. The cells were detached and seeded at a density of 0.5 cells/well on a 96-well plate. After expansion, single cell clones were screened by Sanger sequencing and verified on the protein level by Western blot analysis.

### Polymerase chain reaction

For the extraction of genomic DNA (gDNA) and PCR we used Phire Tissue Direct PCR Master Mix (cat.-nr. F170S, Thermo Fisher Scientific, Waltham, USA) according to the manufacturers´ protocol. The CHD4 alleles were amplified with a previously designed primer pair as mentioned above, receiving an amplicon length of 480 bp. For detailed PCR settings, please refer to Geyer et al.^32^.

### Western blot analysis

Protein expression of the genetically modified cell clones was determined by Western blot analysis. The cells were lysed using cOmplete™ Lysis-M (Roche, Basel, Switzerland) following the manufacturers´ protocol. The protein concentration was determined photometrically utilizing Pierce™ Protein BCA Assay Kit (Thermo Fisher Scientific). Proteins were denatured at 95 °C and an equal amount of protein loaded into 10% Mini-PROTEAN® TGX™ gels (Bio-Rad Laboratories, Feldkirchen, Germany). After electrophoresis, the protein was blotted onto PVDF membranes using the Trans-Blot Turbo Transfer System (Bio-Rad Laboratories). Membranes were blocked with 5% (w/v) milk powder followed by overnight incubation with the primary antibodies. After washing, the membranes were incubated with the secondary antibody for 1 h. At last, the membranes were treated with Pierce™ ECL Substrate (Thermo Fisher Scientific). The signals were detected with Fusion SL Imager (Vilber Lourmat, Eberhardzell, Germany). The following primary antibodies were used: Anti-CHD4 (mouse) (cat.-nr. ab70469, RRID: AB_2229454, Abcam, Cambridge, United Kingdom) diluted 1:2800; Anti-GAPDH (mouse) (cat.-nr. ab374, RRID: AB_2107445, EMD Millipore, Burlington, Massachusetts, USA) diluted 1:700 served as loading control. As secondary antibody, horseradish peroxidase (HRP)-linked goat anti-mouse (cat.-nr. G-21040, RRID: AB_2536527, Thermo Fisher Scientific) diluted 1:10,000 was utilized. Densitometric analysis was done using Image Lab 6.1 (Bio-Rad Laboratories). All western blots were performed more than four times using independent protein lysate preparations to validate the reliability of the results.

### Cell irradiation

Cells grown for 48 h on 6-well cell culture plates were placed inside the CIX2 (XStrahl, Suwanne, Georgia, USA) X-ray radiation cabinet (220 kV, 10 mA, 1.89 Gy/min) at room temperature, ensuring that all wells were located inside the 90% max. homogeneity field. Additionally, dosimetric controls were performed to guarantee a homogenous dose distribution. The cells were treated with radiation doses of 2, 4, and 6 Gy, respectively, or left untreated.

### DNA purification and Sanger sequencing

We used *Sanger* sequencing for screening single cell clones. Prior to sequencing, PCR was done as described above. After amplification, PCR products were purified utilizing the QIaquick PCR Purification Kit (cat.-no. 28104, QIAGEN, Hilden, Germany) according to the manufacturers` protocol. The DNA was eluted with nuclease free water. For detailed analysis, the DNA fragments were sent for Sanger sequencing to Eurofins Genomics. The forward primer (5`-GTTTCCTAGACACCTTACTGCCC-3`) was used as the sequencing primer. The resulting chromatograms were examined using ICE Analysis (Synthego, Redwood City, California, USA) and TIDE^57^.

### Cell counting and colony formation assays

For quantification of viable cells, 50,000 cells/6-well were cultivated for 48 h prior to irradiation. Following the radiation treatment, the cells were washed with phosphate-buffered saline (PBS) and the growth medium was changed. After 2 days, the cells were detached and counted in a Neubauer hemocytometer.

Colony formation assay was performed as described previously^58^. Irradiated cells were plated at 350 cells/6-well and grown for 12 days. Cells were then fixed in 100% methanol for 20 min and stained with 0.05% (w/v) crystal violet (Sigma-Aldrich) for 10 min. Colonies were defined as cell clusters containing ≥ 50 cells and counted for subsequent calculation of the survival fraction as reported previously^58^. All assays were performed in duplicates and repeated in five independent experiments.

### Statistical analysis

Statistical analysis was performed with GraphPad Prism 9 software (GraphPad, San Diego, California, USA). Data sets with two groups were analyzed using the unpaired t-test; data sets with three or more groups were analyzed using the one-way ANOVA and the post-hoc Tukey test. Overall survival (OAS) in the clinical data set was calculated using the Kaplan–Meier method. The survival distributions were compared utilizing the log-rank test. The time from diagnosis to death by any cause was defined as OAS. Quantitative data are given as mean ± standard error of the mean (SEM). Statistical significance was considered at *p*-values < 0.05.

### Ethical approval and consent to participate

The human HNSCC cell line (HN cells, ACC 417) utilized in this research was obtained from DSMZ (Braunschweig, Germany), which is a commercially available cell line, and therefore, informed consent from patients is not applicable in this context and patient identifier and personal information have been removed from this product. Our study has been conducted in accordance with the ethical guidelines and regulations set forth by our institution.

### Supplementary Information

Supplementary Information.

## Data Availability

The data used and analyzed during the current study are available from the corresponding author upon reasonable request.
