# Supplementary material for: CHD4 acts as a prognostic factor and drives radioresistance in HPV negative HNSCC
Source: Sci Rep. 2024 Apr 9;14:8286. doi: 10.1038/s41598-024-58958-z (PMC11003975; doi:10.1038/s41598-024-58958-z)
Supplement: Supplementary file 1 — Supplementary Information. [file 41598_2024_58958_MOESM1_ESM.pdf]

CHD4 acts as a prognostic factor and drives radioresistance in HPV negative HNSCC

Fabian Geyer, Maximilian Geyer, Ute Reuning, Sarah Klapproth, Klaus-Dietrich Wolff, Markus Nieberler

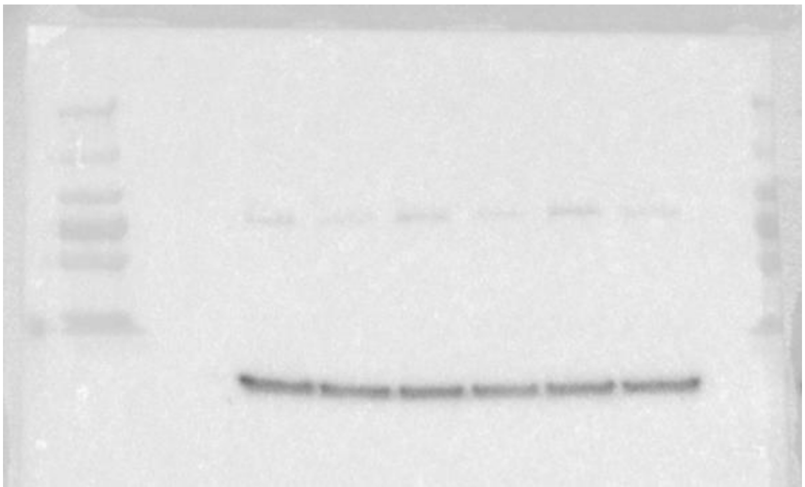

Figure S1. Original CHD4 blot. Original uncropped blot as displayed in Figure 3A. Protein expression of CHD4 in HN WT and HN CHD4 KD cells as determined by Western blot analysis. GAPDH was used as control for equal loading and blotting efficiency. Protein samples of three different experiments were loaded onto one gel and blotted.

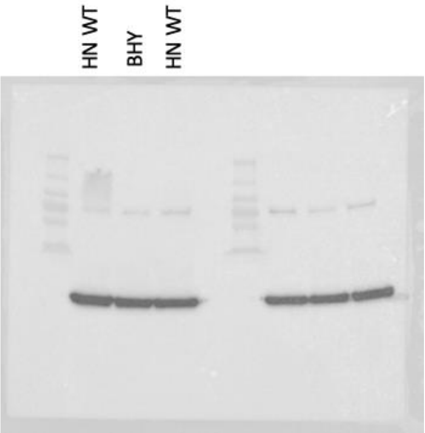

Figure S2. CHD 4 expression in HN and BHY cells. Original uncropped blot of CHD4 expression in HN and BHY cells. GAPDH was used as control for equal loading and blotting efficiency.

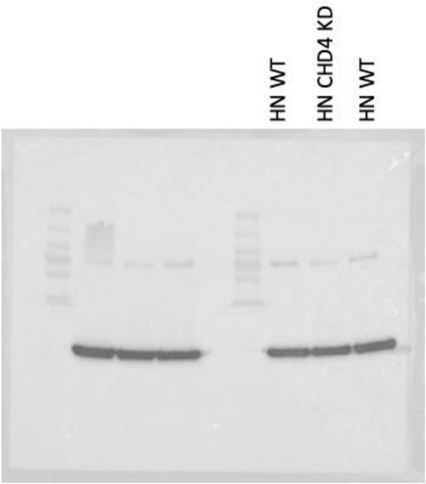

Figure S3. Maintenance of CHD4 knockdown efficiency in HN CHD4 KD cells. Original uncropped blot of the protein expression of CHD4 in HN WT and HN CHD4 KD cells as determined by Western blot analysis. GAPDH was used as control for equal loading and blotting efficiency. HN CHD4 KD cell passage number 42.
